# Influence of Microfluidization Process and Oil Type on the Physicochemical Properties of Nanoemulsions and Their Films

**DOI:** 10.3390/polym18060717

**Published:** 2026-03-16

**Authors:** Hewa Pathiranage Dilani Thilanka Hewa Pathirana, Anna Zimoch-Korzycka, Dominika Kulig, Anna Maria Krawczyk, Shima Vahedi, Magdalena Zyzak

**Affiliations:** 1 Department of Functional Food Products Development, Faculty of Biotechnology and Food Science, Wroclaw University of Environmental and Life Sciences, Chelmonskiego 37, 51-630 Wrocław, Poland; anna.zimoch-korzycka@upwr.edu.pl (A.Z.-K.); dominika.kulig@upwr.edu.pl (D.K.); anna.krawczyk2@upwr.edu.pl (A.M.K.); shima.vahedi@upwr.edu.pl (S.V.); 2 Department of Experimental Biology, Institute of Biology, Faculty of Biology and Animal Science, Wrocław University of Environmental and Life Sciences, C. K. Norwida 25, 50-375 Wrocław, Poland; magdalena.zyzak@upwr.edu.pl

**Keywords:** coconut testa oil, microfluidization, nanoemulsions, edible films, sodium alginate

## Abstract

This study aimed to evaluate the influence of microfluidization cycles and oil type on the physicochemical characteristics of nanoemulsions and the properties of alginate-based edible films. Two types of oil (1%), coconut oil and coconut testa oil, were used for nanoemulsion preparation with Tween 80 and Span 20 (3:2). The emulsions were processed using different numbers of microfluidization cycles (0, 1, 2, and 3) and subsequently mixed with 2% sodium alginate in a 1:1 ratio to obtain film-forming solutions. The film-forming solution containing testa oil showed a particle size of 135.60 ± 37.87 nm, zeta potential of −22.14 ± 3.09 mV, whiteness index of 79.92 ± 2.20, and a creaming index of 0%. These systems produced flexible edible films with significantly (*p* < 0.05) higher elongation at break (1.35 ± 0.17%) and puncture force (2.40 ± 0.32 N), as well as lower water vapor permeability (4.7 × 10^−7^ ± 0.56 × 10^−7^ g m^−1^ h^−1^ Pa^−1^). Fourier Transform Infrared (FTIR) spectroscopy and Scanning Electron Microscopy (SEM) analyses indicated that both the number of microfluidization cycles and the type of oil significantly (*p* < 0.05) influenced the structural and physicochemical characteristics of the resulting edible films.

## 1. Introduction

The development of nanoemulsion-based delivery systems has emerged as a significant area of interest in contemporary food science due to their capacity to encapsulate lipophilic bioactive compounds, enhance oxidative stability, improve bioavailability, and enable controlled release. Nanoemulsions stabilized with biopolymers such as sodium alginate offer additional advantages, including biocompatibility, biodegradability, and suitability for use in foods and edible packaging [1,2]. The choice of lipid phase plays a particularly important role in determining not only the stability of nanoemulsions but also the functional behavior of derived nanoemulsions and edible films. Coconut oil, composed predominantly of saturated medium-chain fatty acids with approximately 50–55% lauric acid (C12), exhibits high oxidative stability and a long shelf life due to its high proportion of saturated lipids (93%) [3,4]. In contrast, testa oil—obtained from the brown outer skin of the coconut kernel and representing a valuable by-product of the coconut processing industry—contains 40–50% oil with significant amounts of lauric acid (32–40%). Although its slightly higher palmitic acid content (11–14%) contributes to faster oxidation compared with coconut oil, testa oil is substantially richer in polyphenolics, tocopherols, and phytosterols [3,5,6], making it an attractive candidate for functional food and therapeutic applications.

Beyond the role of lipid composition, the formulation and stability of nanoemulsions depend strongly on the type and concentration of emulsifiers, their hydrophilic–lipophilic balance (HLB), and the homogenization method used during processing [7,8,9]. Reducing droplet size to the nanoscale is a critical factor that enhances stability, optical clarity, and resistance to destabilization phenomena such as creaming, flocculation, and gravitational separation [10,11,12,13,14]. Among available emulsification techniques, microfluidization has gained prominence as a high-energy, highly scalable method capable of generating nanodroplets with controlled size distribution, low polydispersity, and high physical stability. During this process, coarse emulsions are forced through microchannels at high pressures (3.45–137.90 MPa, in case of industrial scale even >200 MPa), creating intense shear forces that effectively disrupt droplets; parameters such as pressure and the number of cycles directly determine the final droplet size. Nanoemulsions exhibit higher stability than conventional emulsions, mainly due to their small droplet size (typically 20–200 nm). Smaller droplets reduce gravitational separation (creaming or sedimentation), minimize collisions between droplets, and slow down coalescence and Ostwald ripening. In addition, the large surface-to-volume ratio allows surfactants to form a more uniform and durable interfacial layer around each droplet, increasing kinetic stability. As a result, nanoemulsions retain their physicochemical properties for a longer period of time compared to conventional emulsions [11,12].

These characteristics make microfluidized nanoemulsions particularly attractive for food applications, where optical clarity, stability during storage, and compatibility with various matrices are essential. Previous studies have demonstrated their applicability in beverages, dairy products, and meat systems with only minor formulation modifications [15,16,17]. Beyond their formation, the incorporation of nanoemulsions into edible and biodegradable films represents a promising strategy for developing advanced food packaging materials. Sodium alginate, known for its excellent film-forming capacity, can generate flexible, semi-permeable matrices whose barrier, mechanical, and optical properties may be further improved through the incorporation of nanoemulsified oils. The inclusion of functional lipids may additionally impart antioxidant or antimicrobial functionality, aligning with current trends in sustainable and active food packaging that enhance shelf life while reducing reliance on synthetic food preservatives. A key technological aspect of this study is the use of microfluidization not only to produce stable nanoemulsions but also to enhance the structural and functional properties of alginate-based edible films. This integrated approach enables a comprehensive assessment of how formulation variables—particularly oil type (coconut and testa oil) and the number of microfluidization cycles (0, 1, 2, and 3)—affect the physicochemical characteristics of both the emulsions and the resulting films. By examining parameters such as droplet size, zeta potential, pH, electrical conductivity, whiteness index, film thickness, mechanical performance, solubility, and water vapor permeability, the study provides a holistic understanding of the structure–function relationships underlying nanoemulsion-based edible films.

Even though coconut oil has been extensively studied as a lipid carrier in emulsion systems for cosmetic, food, and pharmaceutical industries, detailed characterization of coconut testa oil in emulsion systems have received limited scientific attention. The research gap covered by this study is that it explores underutilized coconut testa oil as a functional ingredient of colloidal delivery systems and edible film formulations. Therefore, the aims of this study were to investigate the effect of coconut testa oil on the physicochemical and functional properties of emulsions and films, and to determine how oil type and the number of microfluidization cycles influence the resulting film properties.

## 2. Materials and Methods

### 2.1. Materials and Chemicals

Tween^®^ 80 (Hydrophilic Lipophilic Balance (HLB) = 15), Span^®^ 20 (HLB = 8.6), glycerol, and sodium alginate (A1112, low viscosity, brown algae, Lot #SLCG0203) were obtained from Sigma-Aldrich (St. Louis, MO, USA). Ultra-pure water (Pol-water, Warsaw, Poland) was used as an aqueous medium for nanoemulsion preparation. As a dispersed phase, coconut oil and testa oil were used. The coconut oil was obtained from BIO FOOD (Ciechocin, Poland). The testa oil was imported from the Coconut Research Institute (Lunuwila, Sri Lanka).

### 2.2. Preparation of Nanoemulsion

The 1% oil (coconut oil/testa oil), and 2% emulsifiers of a mixture of Tween^®^ 80 and Span^®^ 20 in the ratio of 3:2 (HLB value 12.44) were mixed with ultra-pure water followed by low-pressure homogenization (IKA^®^ Ultra-Turrax T25 homogenizer and S25 EC-C-18 G dispersing tool, IKA-Werke GmbH, Staufen, Germany) at 15,000 rpm for 5 min to make a coarse emulsion. The coarse emulsion was subjected to the Microfluidic^®^ machine (Microfluidics M-110 P, Microfluidics International Corporation, Westwood, MA, USA) to prepare a nanoemulsion with three cycling processes (1, 2 and 3 cycles). A blend of Tween^®^ 80 (HLB 15) and Span^®^ 20 (HLB 8.6) was selected to obtain a target HLB value of 12.44, optimized for stabilizing coconut and testa oil in O/W nanoemulsion systems. The combination of non-ionic surfactants enhances interfacial packing and reduces interfacial tension more efficiently than single-surfactant systems, particularly under high-pressure microfluidization conditions (200 MPa). The coarse emulsion was used as a control (cycle 0). The temperature of the samples, 8 °C ± 3 °C, was maintained during the emulsification process. The sodium alginate stock solution (2% *w*/*v*) was first prepared and mixed with glycerol (20% of the dry weight of polysaccharide) at 300 rpm for 30 min (IKA^®^ C-MAG HS7, IKA-Werke GmbH, Staufen, Germany). After each microfluidization cycle, the nanoemulsion was combined with the alginate stock solution at a 1:1 ratio, followed by stirring at 300 rpm for 30 min. This dilution step yielded a final sodium alginate concentration of 1% in the film-forming mixtures. Eight different treatments were prepared, as shown in Table 1.

### 2.3. Preparation of Edible Film

The prepared nanoemulsions (Table 1) were used as film forming solutions. The solution (70 mL) was poured into a Teflon casting plate (27.5 cm × 11 cm) and dried in a chamber (60% relative humidity, 20 °C) (BINDER KBF115UL-240V climatic chamber, BINDER GmbH, Tuttlingen, Germany) for 48 h.

### 2.4. Characteristics of Nanoemulsion

#### 2.4.1. Particle Size and Zeta Potential

The particle size (average droplet diameter) and zeta potential were analyzed through a dynamic light scattering analyzer (Zetasizer PRO, Malvern Panalytical, Malvern, UK) directly for the film-forming solution. The average droplet diameter was expressed as the Z-average obtained by cumulant analysis (intensity-weighted mean). Measurements were performed after a 120 s equilibration at 25 °C.

#### 2.4.2. Whiteness Index

A Chroma meter (MINOLTA CR-400 and CR-A33d Light Projection Tube (ø 22 mm disc, Konica Minolta, Osaka, Japan) was used to measure the color of the film-forming solution. The calibration white plate of CR-A33a Konica Minolta, Osaka, Japan, was used with the coefficients of Y = 93.8, x = 0.3133, and y = 0.3195. The whiteness index (WI) of the film-forming solution was analyzed based on the following calculation (Equation (1)) [18] through the measurement of the CIE L* (lightness), a* (red–green), and b* (yellow–blue) parameters.

(1) $$WI=100-\sqrt{{\left(100-L*\right)}^{2}+({a*}^{2}+{b*}^{2})}$$

#### 2.4.3. Spectrum Analysis

The UV absorbance spectrum of film-forming solution was measured using a UV-Vis Spectrophotometer (UV line 9400 SI Analytics, Mains, Germany) from 190 nm to 400 nm. The distilled water was the blank sample for each spectrum analysis. The film samples of equal thickness were put in the path of light, and the absorbance was recorded. An absorbance at 600 nm (A600) was used to analyze the opacity of the films.

(2) $$\mathrm{Opacity}=\frac{\mathrm{Absorbance}\;\mathrm{of}\;600\;\mathrm{nm}\;}{\mathrm{Thickness}\;\mathrm{of}\;\mathrm{the}\;\mathrm{film}\;(\mathrm{mm})}$$

#### 2.4.4. Determination of the Creaming Index

The creaming index (CI) of the film-forming solution was analyzed after 40 days of storage at 4 °C ± 2 °C. Samples (100 mL) were placed in graduated cylindrical glass tubes and stored vertically. After storage, the total emulsion height (HE) and the height of the separated upper layer (HC) were measured using a digital caliper (±0.01 mm) under standardized illumination conditions with adjustable LED backlighting to enhance phase boundary visibility. Measurements were performed in triplicate. The CI was calculated according to Equation (3) [19].

(3) $$\mathrm{Creaming}\;\mathrm{Index}\;(\mathrm{CI})\%\;=\;\frac{\mathrm{Height}\;\mathrm{of}\;\mathrm{the}\;\mathrm{cream}\;\mathrm{layer}\;(\mathrm{HC})}{\mathrm{Hight}\;\mathrm{of}\;\mathrm{the}\;\mathrm{emulsion}\;(\mathrm{HE})}\times 100\%$$

#### 2.4.5. Rheological Analysis

##### Flow Property

The flow properties of film forming solution were analyzed using a HAAKE RheoStress 6000 rheometer (Thermo Scientific, Karlsruhe, Germany) according to the method described by Kulig et al., 2017 [20]. The measuring device was driven using RheoWin Job Manager version 4.00 software. The shear stress (τ) and viscosity (η) measurements were obtained in a two-step analysis: (1) at a constant shear rate of 5 s^−1^ within 2 min and (2) under a controlled shear rate of 600–7760 s^−1^ within 2 min (20 °C). The results obtained from the second step were used to show flow curves.

##### Thixotropy

Thixotropy was tested according to method described by Kulig et al., 2017 [20]. The Newtonian or non-Newtonian behavior of emulsions and their recovering capability were analyzed over time using ramped-up and ramped-down flow curves. The hysteresis loop was obtained in 3 steps: increasing the shear rate from 0 s^−1^ to 100 s^−1^ for 2 min, followed by maximum shear rate 100 s^−1^ for 1 min, and decreasing the shear rate from 100 s^−1^ to 0 s^−1^ in 2 min. The area of the loop was calculated as a thixotropic area using RheoWin Data Manager software version 4.00 (Thermo Scientific, Karlsruhe, Germany).

##### Viscoelastic Properties

Viscoelastic properties were tested according to method described by Kulig et al., 2017 [20]. Analyzes were preceded by a strain sweep (0.001–10 Pa) at constant frequency 1 Hz to determine linear viscoelastic region. The viscoelastic properties were then evaluated using oscillatory shear test over a range of oscillation frequencies (0.01–10 Hz) at a constant strain oscillation amplitude (0.01 Pa) and temperature (20 °C). The storage modulus (G′), loss modulus (G″), and loss tangent (δ) were presented as mean values measured at 1 Hz.

### 2.5. Evaluation of the Quality of the Film

#### 2.5.1. Tensile Strength

The tensile strength of the sample was analyzed using a Zwick/Roell Z010 tensile strength machine with 8033 endings using the method of ASTM D882-12. *Standard Test Method for Tensile Properties of Thin Plastic Sheeting.* ASTM International: West Conshohocken, PA, USA, 2012. The film sample was cut based on the ISO 527-2:2012.Plastics-Determination of tensile properties-Part 2: Test conditions for moulding and extrusion plastics. International Organization for Standardization: Geneva, Switzerland, 2012. The films were stretched using a crosshead speed of 125 mm/min. The tensile strength (σ) of the film was calculated as force per unit area (MPa). The ratio between the changed length and initial length was taken as elongation at break/strain at tensile strength (ε) (%).

#### 2.5.2. Puncture Testing

The puncture test of the film was analyzed using a ZwickRoell Z010 machine. The test sample (3 cm × 3 cm) was used based on the ASTM F 1306 test standard (Standard Test Method for Slow Rate Penetration Resistance of Flexible Barrier Films and Laminates). The speed of the test was 25 mm/min. The maximum force of puncture (N) was recorded.

#### 2.5.3. Water Vapor Permeability

The water vapor permeability of films was determined gravimetrically using a method described by Zimoch-Korzycka et al., 2015 [21]. The circular film samples were placed on in a glass cup filled with 100 mL of distilled water and sealed with a rubber ring. The weight of the whole was measured as the initial weight. Then, the sample was placed in a chamber at 4 °C and 60% relative humidity (BINDER KBF115UL-240V climatic chamber, BINDER GmbH, Tuttlingen, Germany) for 6 h. After 6 h, the weight of the sample was measured.

The water vapor transmission rate (*WVTR*) of the film sample was calculated using the following equation (Equation (4)), where “*G*” is the weight changes in the cup [g], “*t*” is time (6 h.), and “*A*” is the area of the cup (0.002826 m^2^).

(4) $$WVTR=\frac{G}{t\times A}$$

The water vapor permeability (*WVP*) of the sample was measured using the following equation (Equation (5)), where the “*S*” is water vapor pressure at 4 °C (810 Pa).

“*R*1” is the relative humidity inside the cup (100%) and “*R*2” is the relative humidity of the controlled chamber (60%). The “*d*” is the thickness of the film [m].

(5) $$WVP=[\frac{WVTR}{S\left(R1-R2\right)}]\times d$$

#### 2.5.4. Solubility Measurement

The solubility of the film sample was analyzed using a 3 cm × 3 cm sample, weighed using an analytical balance with a precision of ±0.0001 g to determine the initial mass (W initial). Then, it was placed in a beaker with 50 mL of deionized water followed by agitation at 200 rpm for 1 h at ambient temperature (25 °C ± 3 °C) (Universal Shaker SM 30 C Edmund Bühler GmbH, Bodelshausen, Germany). The solution was filtered through initially dried filter paper. Then insoluble particles are dried with filter paper at 60 °C until they reach a constant weight. The dried residue was calculated (W residue). The solubility of the sample was calculated using the following equation (Equation (6)).

(6) $$Solubility\;\left(\%\right)=\frac{W\;initial-W\;residue}{W\;initial}\times 100$$

#### 2.5.5. Fourier Transform Infrared Spectrometry Analysis

Infrared spectra were analyzed for each film treatment and pure sodium alginate 1% film (Shimadzu spectrometer, ATI Mattson, Kyoto, Japan). The absorbance spectra were collected at 2 cm^−1^ resolution and by 64 scans, directly on films with a Golden Gate ATR accessory (Specac Ltd., Orpington, UK).

#### 2.5.6. Scanning Electron Microscopy

The edible films prepared from each type of nanoemulsion were analyzed through a scanning electron microscope, Zeiss EVO 15 LS scanning electron microscope (Carl Zeiss AG, Oberkochen, Germany), with 500× magnification. The samples were firstly sputtered with a layer of gold in an SC7620 sputter coater (Edwards Ltd., Crawley, UK). Digital visualization of images was performed in cooperation with the Electron Microscopy Laboratory at the Wrocław University of Environmental and Life Sciences.

### 2.6. Statistical Analysis

Statistica 14 software (TIBCO Software Inc., Palo Alto, CA, USA) was used for all statistical analyses. Experimental data were analyzed using a factorial ANOVA (two-way ANOVA) model to evaluate the significance of: (1) the main effects of each factor (oil type and number of microfluidization cycles) and (2) their interaction effect. When significant differences were detected (*p* < 0.05), mean separation was conducted using Tukey’s post hoc test. Data are presented as mean (*n* = 3) ± standard deviation, and graphical outputs were prepared using Microsoft Excel 365 (Microsoft Corp., Redmond, WA, USA). The experiment was performed in triplicate, and all analyses were also run in triplicate.

## 3. Results and Discussion

### 3.1. Particle Size

Emulsion systems are commonly described according to their droplet size; however, size alone does not strictly define their classification. While macroemulsions typically exhibit droplet diameters above 1 μm and nanoemulsions generally range from approximately 20 to 500 nm, the distinction between nanoemulsions and microemulsions is primarily based on thermodynamic stability rather than size. Microemulsions are thermodynamically stable systems formed spontaneously, whereas nanoemulsions are kinetically stable systems produced using external energy input, such as high-pressure homogenization [10]. Reducing droplet size into the nano-range is a critical factor influencing the physical stability, optical properties, and functional performance of emulsions. Smaller droplets exhibit lower gravitational separation, reduced creaming and flocculation, and improved kinetic stability, making nanoscale systems particularly advantageous for food and packaging applications [13].

A significant influence of the number of microfluidization cycles, as well as the interaction between oil type and cycle number, was observed for particle size (Table 2). The droplet diameter of the nanoemulsions decreased progressively with each additional cycle, from 556.18 ± 63.83 nm in the coarse emulsion to 127.01 ± 33.44 nm after three cycles. This trend is consistent with previous findings, in which a nanoemulsion containing 10% oil (50% fish oil and 50% lemon oil), 1% of Tween 80, and 1% sodium alginate (pH = 3) reached a particle size of approximately 132 nm after three microfluidic passes [7]. Similar to that study, the reduction in droplet size was most pronounced up to the third cycle, supporting our decision not to evaluate higher cycle numbers.

Preliminary analyses performed before mixing the emulsions with the sodium alginate solution (2%) showed that the particle size of the pure coarse emulsion decreased from 166.68 nm to 38.44 nm after three microfluidization cycles. After incorporation into the film-forming solution, an increase in apparent particle size was observed. This increase does not necessarily indicate droplet coalescence, but may reflect changes in the hydrodynamic diameter measured by DLS due to interactions between alginate and the oil droplets. Anionic polysaccharides such as sodium alginate can adsorb onto droplet surfaces, increasing the effective hydrodynamic radius, or induce bridging or depletion flocculation depending on polymer concentration and interfacial affinity [22,23,24]. Similar increases in apparent droplet size after polysaccharide addition have been reported in emulsion–biopolymer systems, where polymer–droplet interactions modify dispersion structure without necessarily causing irreversible aggregation [22,23]. Importantly, no evidence of macroscopic phase separation was observed in the optimized systems (CI = 0% after ≥2 cycles), suggesting that the observed size increase reflects structural reorganization within the nanoemulsion–polymer matrix rather than instability.

Nanoemulsion–biopolymer systems with droplet sizes below 200 nm offer functional advantages, as their higher surface-area-to-volume ratio enhances the release and potential absorption of lipophilic bioactive compounds during digestion [13]. This demonstrates the relevance of controlling droplet size through microfluidization when designing nanoemulsion-based edible films with targeted functional properties.

**Figure 1:**
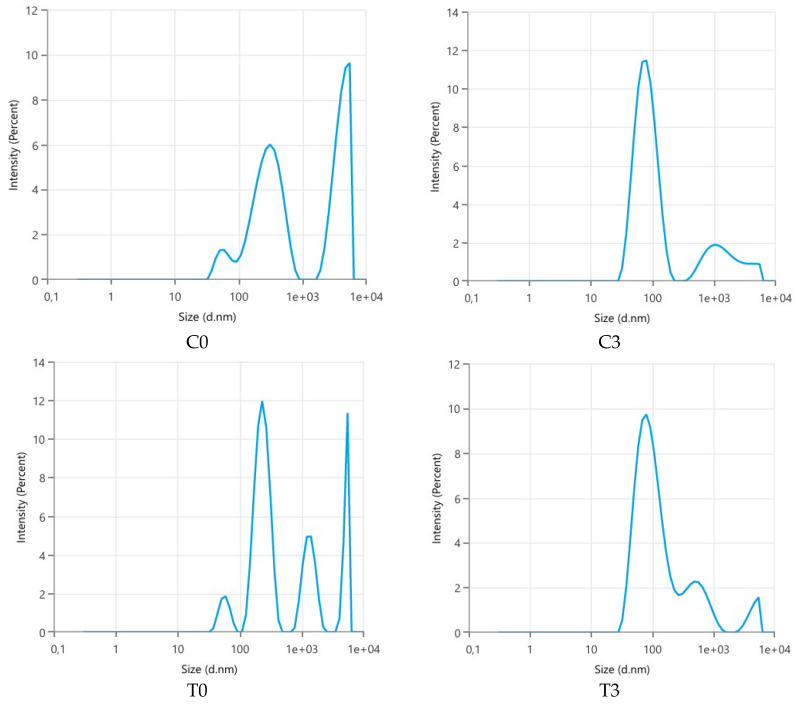
Size distribution by intensity of coarse emulsion with coconut oil (C0), nanoemulsion with coconut oil after 3rd microfluidic cycle (C3), coarse emulsion with testa oil (T0), nanoemulsion with testa oil after 3rd microfluidic cycle (T3).

Figure 1 illustrates the size distribution patterns across the examples of untreated and treated emulsions through microfluidization processes (C0, C3, T0, T3), showing how intensity varies with particle or object size in each case.

The polydispersity index (PDI) provides additional insight into the uniformity of droplet size distribution within the nanoemulsions. As shown in Table 2, microfluidization had a clear effect on improving size homogeneity, with PDI values decreasing progressively with the number of cycles for both oils. Coarse emulsions exhibited the highest PDI values (0.77–0.82), indicating broad droplet-size distributions typical of insufficiently homogenized systems. After three microfluidization cycles, the PDI was reduced to 0.30–0.31, reflecting a narrower distribution and more uniform droplet population.

These reductions in PDI correspond well with the observed decreases in mean particle size and the increasingly negative zeta potential values, collectively suggesting improved colloidal stability and more consistent droplet packing. A lower PDI reflects a narrower size distribution and higher monodispersity of the system, which is commonly associated with enhanced colloidal stability. Moreover, more negative zeta potential values indicate stronger electrostatic repulsion between droplets, reducing the likelihood of aggregation [25]. Although coconut and testa oil differed slightly in their initial PDI values, both responded similarly to microfluidization, indicating that the homogenization process dominated over the intrinsic oil-type effects in governing droplet-size uniformity.

### 3.2. Zeta Potential

The individual effects of oil type, microfluidization cycles, and their interaction significantly (*p* < 0.05) influenced the zeta potential of the nanoemulsions (Table 2). Coconut oil formulations exhibited the lowest zeta potential (−19.36 mV), which may be attributed to stronger interaction between medium-chain saturated fatty acids (particularly lauric acid) and the negatively charged COO^−^ groups of sodium alginate [26]. In contrast, testa oil contains a higher proportion of long-chain fatty acids (such as oleic acid), which interact less effectively with the sodium alginate COO^−^ groups; as a result, formulations containing testa oil showed comparatively higher zeta potential values (−17.54 mV). Previous studies have demonstrated that nanoemulsions with zeta potential values greater than +30 mV or less than −30 mV tend to exhibit strong electrostatic repulsion and therefore greater stability [27,28].

For both oils, the zeta potential shifted progressively from values near −13 mV in the coarse emulsions to values approaching −17, −23, and −24 mV with increasing microfluidization cycles (Table 2). Although these values do not exceed the commonly cited ±30 mV threshold for highly stable systems, the increasing magnitude of ζ-potential suggests enhanced electrostatic repulsion between droplets, thereby reducing their tendency to flocculate and contributing to improved kinetic stability [27,28].

The observed trend aligns with the reduction in droplet size induced by microfluidization. This is consistent with the findings of Prakash and Parida (2023) [29], who reported a decrease in zeta potential from −10.6 ± 2.1 mV to −14.3 ± 2.1 mV as particle size decreased. Reduction in droplet size increases the interfacial surface area available for interaction with the negatively charged COO^−^ groups of sodium alginate, resulting in increasingly negative zeta potential values. Although the absolute values did not cross the ±30 mV boundary, the progressive increase in surface charge magnitude, combined with steric stabilization from emulsifier and the alginate network, contributed to the overall stability of the system. This explains why the nanoemulsions remained stable despite their moderately negative charge, and is further supported by the creaming index results.

The increase in the negative zeta potential observed after successive microfluidization cycles suggests a higher degree of dissociation of the alginate COO^−^ groups, which enhances both the solubility and dispersion of the polymer chains in the continuous phase. More negatively charged alginate chains experience stronger electrostatic repulsion, preventing aggregation and maintaining a well-solubilized matrix. At the same time, the greater availability of dissociated COO^−^ groups can subsequently facilitate more efficient ionic cross-linking with Ca^2+^ ions, improving the gel-forming ability of the system once cations are introduced.

### 3.3. pH and Conductivity

The pH and electrical conductivity (EC) of the film-forming nanoemulsions did not differ significantly (*p* < 0.05) among the treatments, with average values of 5.55 and 2037.53 µS/cm, respectively. Although higher pH values closer to neutral conditions generally promote more negative zeta potentials and therefore greater electrostatic stability [30], the pH range observed in this study remained relatively constant and did not contribute to differences in droplet charge. The positive EC values reflect the presence of ionic species dissolved in the continuous phase, which is typical for emulsions containing surfactants, biopolymers, and small electrolyte residues. Similar relationships between electrical conductivity and emulsion composition have been reported by Da Costa et al. (2014) [31].

### 3.4. Transparency, Whiteness Index and Spectrum

The use of edible coatings in food applications is largely determinated by their optical, mechanical, and functional properties, as well as their ability to maintain or mimic the natural appearance of the product. Coating materials should be sufficiently transparent or visually compatible with the surface of the food, enhancing appearance without masking defects of the products [32].

**Figure 2:**
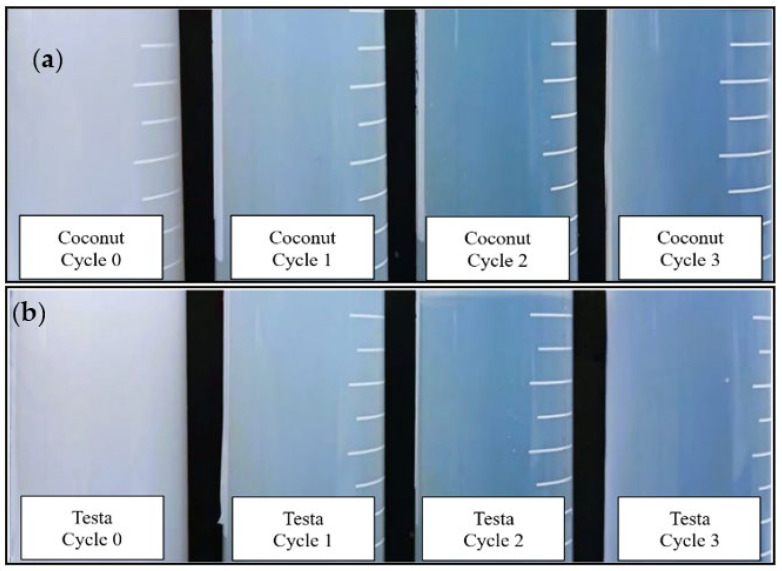
Visual observation of coconut oil (**a**) and testa oil (**b**) film-forming solutions produced with progressive microfluidic cycles (0, 1, 2, 3).

In the present study, the transparency of the film-forming nanoemulsions increased with the number of microfluidization cycles (Figure 2), which is consistent with the reduction in droplet size. Emulsions with smaller droplet diameters (particle sizes from 20 nm to 200 nm) scatter less light and therefore appear more transparent, whereas systems with droplet sizes above 200 nm typically exhibit a milky appearance [33,34].

Although the whiteness index (WI) is influenced by optical scattering phenomena, it does not exhibit a simple linear correlation with particle size, as WI is calculated from the L*, a*, and b* color coordinates. In the present study, WI increased despite droplet size reduction, reflecting enhanced optical homogeneity and brightness rather than increased turbidity.

**Figure 3:**
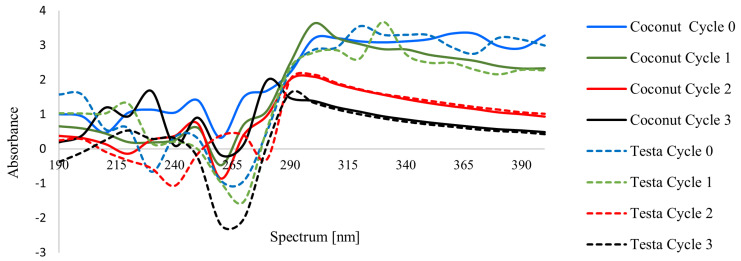
Changes in the spectrum of the film-forming solution during the microfluidic cycling process with different types of oil.

These findings were further examined using UV–Vis spectral analysis (Figure 3). Minor negative absorbance values occasionally appeared due to baseline subtraction artefacts caused by light scattering from the nanoemulsion droplets, rather than true negative absorbance. These values are non-physical and do not affect the interpretation of the spectral trends. The spectral profiles indicated that the influence of microfluidization on absorbance depended on both the oil type and the number of cycles. A noticeable reduction in absorbance beyond approximately 290 nm was observed only for samples subjected to two and three microfluidization cycles (C2, C3, T2, and T3), indicating improved optical clarity as a result of substantial droplet refinement. In contrast, the control samples (C0, T0) and those processed with a single cycle (C1, T1) did not exhibit a consistent trend; their absorbance curves fluctuated, showing alternating increases and decreases rather than a stable downward pattern. The fluctuating spectral profiles likely reflect enhanced multiple scattering caused by larger droplets and incipient aggregation, which is in line with their higher particle size and polydispersity index, less negative zeta potential and higher creaming index. This suggests that a single microfluidization pass does not sufficiently reduce droplet size to influence light absorption behavior, whereas multiple cycles are required to produce detectable spectral changes. This observation is consistent with the findings of Prakash and Parida (2023), who also reported that the spectral characteristics of coconut oil emulsions enriched with vitamin E were influenced by formulation and processing conditions [29].

Despite these changes observed in the liquid emulsions, the effect of microfluidization on the whiteness index diminished once the films were dried. The whiteness index (average WI = 90.51) and opacity (average 18.89 mm^−1^) of the final edible films did not differ significantly (*p* > 0.05) among treatments, likely due to similar concentrations of oil pigments and volatile compounds in all formulations after solvent evaporation.

### 3.5. Rheological Properties

#### 3.5.1. Flow Properties

Three main factors of Brownian diffusion, surface forces, and hydro-dynamic interaction influence the rheology of nanoemulsions. Additionally, particle size reduction enhances the texture and rheological properties of nanoemulsions [24].

**Figure 4:**
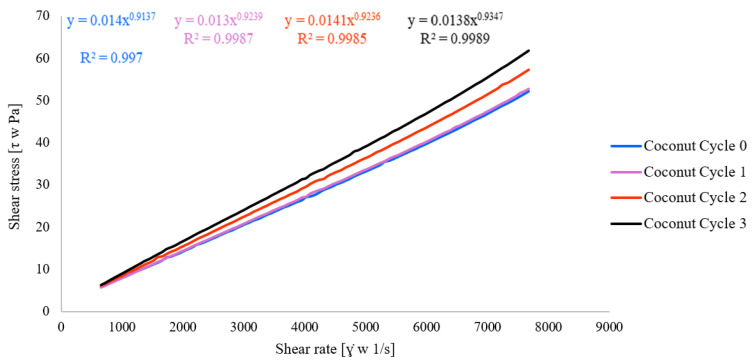
Flow curves for coconut oil nanoemulsion with microfluidic cycles.

**Figure 5:**
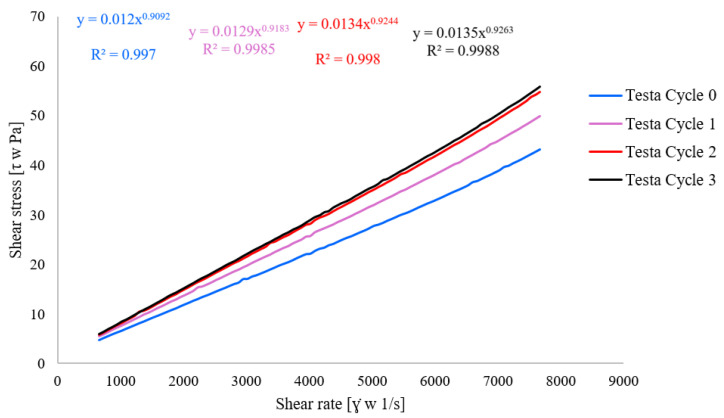
Flow curves for testa oil nanoemulsion with microfluidic cycles.

The flow curves of two types of oil-containing emulsions with microfluidic cycles are presented in Figure 4 and Figure 5.

The fluid exhibits pseudoplastic (shear-thinning) behavior, as its apparent viscosity decreases with increasing shear rate. Such non-Newtonian flow is characteristic of emulsions and colloidal dispersions, where structural rearrangements, droplet alignment, and breakdown of weak flocculated networks occur under shear stress [23,24].

It confirmed that lowering viscosity during microfluidization makes more homogenous samples with smaller molecule structures [35]. This behavior can indicate good physical stability. The shear-thinning emulsions often have structured continuous phases (e.g., due to polysaccharides, proteins, or emulsifiers) that provide stability at rest but allow for easier flow under stress, which is desirable in food, cosmetic, and pharmaceutical applications.

Despite these rheological changes, the apparent viscosity at low (5 s^−1^—average 0.0161 Pa·s) and high (7750 s^−1^—average 0.0069 Pa·s) shear rates did not change significantly (*p* > 0.159) within the main effect and interaction effect. These results further confirmed that, even though microfluidic cycle changes the flow characteristics and structural properties, it does not significantly influence the changes in apparent viscosity at a specific level (5 s^−1^ and 7750 s^−1^).

#### 3.5.2. Thixotrophy

**Figure 6:**
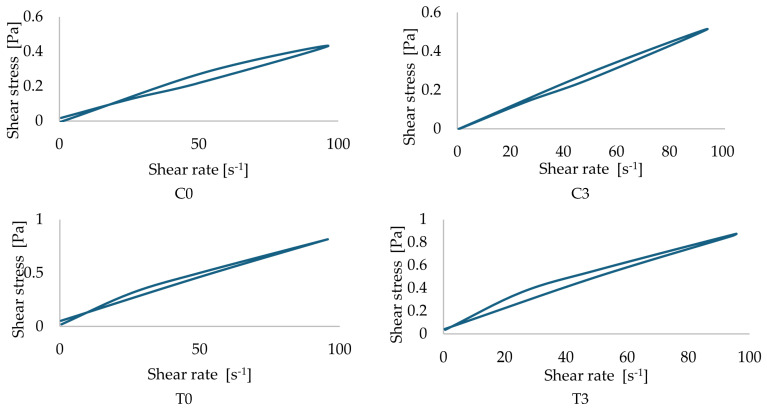
Thixotropy curves of coarse emulsion with coconut oil (C0), nanoemulsion with coconut oil after 3rd microfluidic cycle (C3), coarse emulsion with testa oil (T0), nanoemulsion with testa oil after 3rd microfluidic cycle (T3).

The thixotropy values expressed as the hysteresis loop area in Pa·s of emulsions formulated with coconut oil and testa oil across successive shear cycles (cycle number 0–3) are presented in Table 2. The results indicate distinct differences (*p* < 0.013) in the structural recovery and rheological stability of these emulsions depending on the oil type, microfluidic cycles and the interaction of both. Examples of thixotropic curves of chosen emulsions are presented in Figure 6.

Emulsions with coconut oil exhibited moderate and relatively stable thixotropic values, decreasing gradually from approximately 4 Pa·s to 2 Pa·s over the two cycles of microfluidization. This indicates a good ability to recover their internal structure after shear, suggesting that these emulsions maintain rheological stability under repeated mechanical stress within two microfluidic cycles. In contrast, emulsions formulated with testa oil showed an initially very high thixotropy (~10 Pa·s) in the coarse emulsion, followed by a sharp decrease, reaching negative values in the subsequent second cycle (down to approximately –2 Pa·s). This behavior suggests a rapid and irreversible breakdown of the internal structure, with limited or no ability to rebuild the network after shear. The negative thixotropy values may also indicate non-ideal flow behavior, potentially associated with emulsion destabilization or phase separation [36]. These findings highlight the superior structural resilience of emulsions based on coconut oil compared to those containing testa oil, which appear more susceptible to degradation under shear conditions. The difference in fatty acid composition of both oil types is the main factor for changes in structural recovery within the same shear conditions.

#### 3.5.3. Viscoelastic Properties

Table 2 presents changes in loss tangent (tan δ) measured at 1 Hz over successive shear cycles (Cycle 0–3) for emulsions prepared with coconut oil and testa oil. Loss tangent (tan δ), defined as the ratio of the viscous modulus (G″) to the elastic modulus (G′), serves as an indicator of the relative dominance of viscous versus elastic behavior in viscoelastic systems. Emulsions formulated with coconut oil initially exhibited a very high tan δ (~11), indicating predominantly viscous, fluid-like behavior, suggesting a loosely structured internal network. However, tan δ decreased sharply over the subsequent cycles, reaching values below 1 by the third cycle. This trend suggests a progressive transition toward a more elastic, solid-like structure, possibly as a result of structural reorganization or gelation processes occurring under mechanical shear [36].

In contrast, emulsions prepared with testa oil demonstrated initially low tan δ values (~0.3), reflecting a more elastic and gel-like behavior from the outset. Over the subsequent cycles, tan δ showed a moderate increase (up to ~3), followed by a slight decrease. These changes were less pronounced than in the coconut-based emulsions, indicating greater rheological consistency but also suggesting limited ability for structural adaptation or recovery under repeated shear.

Taken together with the thixotropy results, these findings highlight contrasting structural dynamics. Coconut oil-based emulsions exhibit initial fluidity but develop greater elastic character over time, indicating structural reinforcement and rheological stabilization with repeated deformation.

Testa oil-based emulsions, although initially more gel-like, appear less capable of maintaining or rebuilding structure, as evidenced by earlier breakdown in thixotropic properties.

These differences may be attributed to the molecular composition and interfacial behavior of the oils, which affect droplet interactions, network formation, and the emulsion’s response to mechanical stress [24].

### 3.6. Creaming Index

The creaming index of the film-forming solutions reflected their physical stability during storage. Both oil type and the number of microfluidization cycles significantly affected the creaming index (*p* < 0.05).

It should be noted that the creaming index does not represent the volume fraction of pure oil but rather the relative height of the droplet-concentrated upper layer. Even at low oil content (0.5%), gravitational migration can lead to the formation of a visibly thicker cream layer containing a concentrated dispersion of droplets. Considering the main effect of oil type in the two-way ANOVA, testa oil emulsions exhibited a significantly (*p* < 0.05) higher creaming index than those prepared with coconut oil. However, this difference cannot be attributed solely to fatty acid composition, but rather to oil-dependent physicochemical properties that influence droplet interactions and destabilization pathways [23,37].

Furthermore, the content of lauric acid (26.66–32.04%), myristic (18.31–19.60%), and palmitic acid (13.43–15.71%) in testa oils is different from coconut kernel oils (32–51%, 17–21% and 6.9–14%, respectively) [3,38].

Coconut oil is rich in saturated medium-chain fatty acids (MCFAs), whereas testa oil contains a lower proportion of saturated fatty acids and a higher content of unsaturated fatty acids, including PUFAs. Differences in fatty acid and triacylglycerol composition may influence oil phase properties such as melting behavior, viscosity, density contrast, and interfacial interactions [23]. At low storage temperatures (4 °C), oils richer in saturated fatty acids may exhibit partial fat crystallization, which can modify droplet rigidity and inter-droplet interactions. Depending on the extent of crystallization, this may either promote partial coalescence or enhance resistance to droplet deformation [37,39].

Considering the main effect of oil type in the two-way ANOVA, testa oil emulsions exhibited a significantly (*p* < 0.05) higher creaming index than those prepared with coconut oil. Once droplets reached the nano-range (≥2 cycles), creaming was completely suppressed for both oils (CI = 0%), demonstrating that gravitational separation was effectively minimized regardless of oil type, consistent with the dependence of creaming rate on droplet diameter as described by Stokes’ law [23].

A significant effect of the microfluidic cycle was observed for changes in the creaming index of the film-forming solution due to particle size reduction with the number of microfluidic cycles. The smaller particles generate higher repulsion between two droplets. It provides more possibilities to combine higher concentrations of sodium alginate COO- ends, which gives a higher negative charge to the oil droplet. If this repulsion is higher than the combination of osmotic attraction and van der Waals forces of molecules in the colloidal system, it makes a stable emulsion without creaming index [40]. Furthermore, when the concentration of sodium alginate increases, viscosity may prevent the separation of the molecules. This is further confirmed by Salvia-Trujillo et al. (2016) through identification of the effect of sodium alginate concentration (0.1% to 1%) on the creaming index [7]. One percent (1%) of sodium alginate had a lower creaming index compared to other sodium alginate concentrations. Therefore, a 1% sodium alginate solution was selected for this study.

### 3.7. Evaluation of the Quality of Edible Film

#### 3.7.1. Tensile Properties

Mechanical properties of edible films, such as tensile strength, elongation, and puncture strength, are important because they determine the film’s ability to protect and preserve food during handling and storage. Strong and flexible films help maintain product integrity, prevent damage, and extend shelf life [41].

The tensile strength of film samples did not show a significant (*p* = 0.332) difference between the main effect and interaction effects, and it showed an average value of 13.72 MPa. However, the interaction and main effect significantly (*p* = 0.004) influence the changes in elongation at break in the percentage of each film, as shown in Table 3.

The main effect of testa oil indicates that it has a significantly (*p* = 0.00) higher elongation ability than coconut oil. This is because of a higher concentration of long-chain fatty acids such as linoleic, oleic and palmitic acid in testa oil than in coconut oil. Moreover, long-chain fatty acids make a stronger bond with sodium alginate with fewer microscopic pin holes than medium-chain fatty acids [42]. This eventually increases the mechanical strength of the edible film with testa oil to show a higher elongation at break.

On the other hand, increasing the number of microfluidization cycles significantly enhanced film elongation. This improvement can be attributed to the greater surface area of the oil droplets generated during microfluidization, which promotes the formation of a more compact network with the sodium alginate chains, thereby increasing the film’s mechanical strength. Finally, the interaction between the third microfluidic cycle and testa oil resulted in a higher elongation at break due to the higher interaction points of sodium alginate with long-chain fatty acids.

#### 3.7.2. Puncture Properties

The main effect of microfluidization cycles significantly (*p* = 0.049) influenced the maximum puncture energy (Table 3). This can be attributed to the reduction in particle size with increasing microfluidization cycles, which increases the surface area of the emulsion droplets. The enlarged interfacial area facilitates the dense deposition of sodium alginate polysaccharides without pinholes, resulting in mechanically stronger films. In contrast, the interaction of the oil type and the microfluidic cycle significantly (*p* = 0.027) increased the puncture properties of the film. This is due to the higher concentration of long-chain fatty acids and the smaller particle size, which makes a dense network with sodium alginate. Therefore, the testa oil that underwent the third microfluidic cycle significantly showed (*p* = 0.00) the highest “maximum force of puncture” in this study.

#### 3.7.3. Water Vapor Permeability

The permeability of an edible film is a key property related to the ability of water molecules to diffuse through the polymer matrix. The type of oil significantly (*p* = 0.005) influenced the water vapor permeability (WVP). Testa oil exhibited significantly lower WVP than coconut oil. This difference may be associated with variations in fatty acid chain length and degree of unsaturation, which can influence lipid–polymer interactions and the structural organization of the dispersed lipid phase within the alginate matrix, thereby influencing barrier performance. Incorporation of lipids into hydrophilic polysaccharide films is widely reported to reduce WVP by increasing film hydrophobicity and creating a more tortuous pathway for water diffusion [43]. Lipid chain length and molecular structure may affect the packing density and compatibility of lipid domains within the polymer matrix, thereby influencing barrier performance [23]. The lower WVP observed for testa oil films suggests a more effective barrier formation within the alginate matrix compared to coconut oil.

The microfluidic cycles encourage a higher surface area to make strong bonds between oil droplets and sodium alginate COO^−^. Therefore, the WVP of T3 film (testa oil, third microfluidic cycle) resulted in a significantly lower permeability value (0.47 × 10^−6^ g m^−1^ h^−1^ Pa^−1^) than other treatments.

On the other hand, Ahmed et al. (2016) [44] confirmed that the incorporation of essentials into the edible film with polylactide makes a microhole in the film matrix, which encourages greater water movement through the film matrix. Moreover, the oil-containing film matrix acts as a laminating cover for edible film, which gives greater protection.

The thickness of the film directly influences the changes in water vapor permeability, tensile strength and puncture properties of edible films. The thickness of the film can be changed with the binding behavior of sodium alginate polysaccharides to fatty acid chains to make a uniform dense matrix. However, the main effect and interaction effect did not influence the changes in the thickness of edible films, which showed a 0.079 mm average thickness.

#### 3.7.4. Solubility

A significant (*p* < 0.05) main effect and interaction effect were observed for the solubility of the films (Table 3). Testa oil films showed significantly (*p* = 0.00) higher solubility than coconut oil films, which may be attributed to the higher proportion of polar compounds present in testa oil. Arivalagan et al. (2018) reported that coconut testa contains a considerable amount of polyphenolics (167 mg GAE/g), including major phenolic acids (protocatechuic, p-coumaric, ferulic acid) and flavonoids such as catechin, apigenin, and kaempferol [45]. In addition, the higher content of unsaturated fatty acids in testa oil may further contribute to increased solubility compared with coconut oil [45,46]. Furthermore, the major phenolics of testa oil are protocatechuic acid, p-coumaric acid and ferulic acid, and major flavonoids are catechin, apigenin and kaempferol. Furthermore, the higher unsaturated fatty acid content of testa oil further accelerates the solubility free fatty acids compared to coconut oil [46].

Importantly, solubility is a key functional parameter for edible films, as it determines their behavior in aqueous environments, including hydration, disintegration, and the release of active compounds during food application or digestion. Higher solubility facilitates faster dissolution of the film matrix and can enhance the availability of encapsulated ingredients, whereas lower solubility contributes to stronger structural integrity and slower release kinetics.

Microfluidization had a significant effect on film solubility, with higher cycle numbers reducing solubility. As the number of cycles increased, droplet size decreased and surface area increased, promoting stronger interactions between sodium alginate chains and resulting in a denser polymer network. Films formed from emulsions with smaller particle sizes therefore exhibited lower solubility, while those with larger droplets allowed greater water penetration and higher dissolution.

A significant interaction between oil type and microfluidization cycle was also observed, with the highest solubility occurring in films prepared from coarse-emulsion testa oil. In addition, as sodium alginate and plasticizers are hydrophilic polymers, they naturally contribute to high dissolution levels [47]. Consequently, all films exhibited solubility values above 80%, supporting rapid hydration and efficient release of functional compounds during food application.

#### 3.7.5. Fourier Transform Infrared (FTIR) Spectrometry 

**Figure 7:**
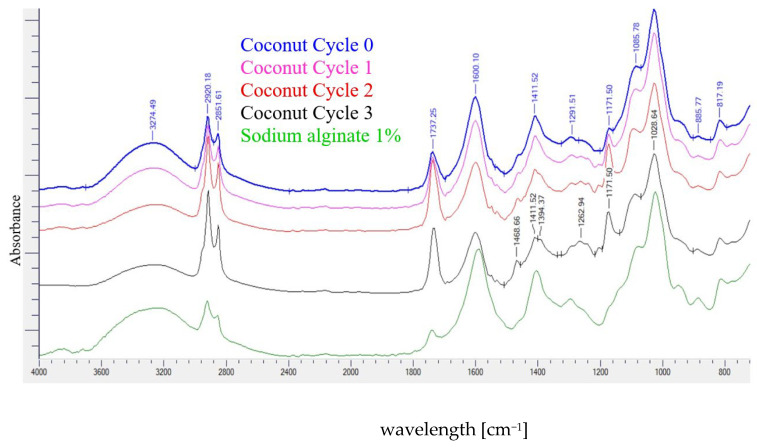
FTIR spectrum of coconut oil-added films with microfluidic cycles.

**Figure 8:**
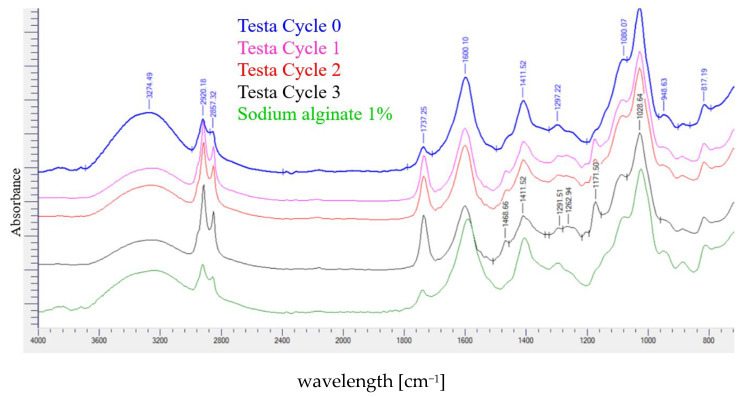
FTIR spectrum of coconut testa oil added films with microfluidic cycles.

FTIR spectra of the coconut and testa oil films with different microfluidization cycles are presented in Figure 7 and Figure 8, respectively. Minor spectral variations were observed between treatments, which may indicate subtle chemical alterations and molecular interactions within the film matrix. Any potential chemical modifications, however, remain speculative and would require verification with additional analytical methods.

The broad peak between 3000 and 3800 cm^−1^, attributed to OH stretching of water molecules in the edible films, remained essentially unchanged across treatments. Slight changes in the peaks at 2920.18 cm^−1^ and 2851.61 cm^−1^ (symmetrical and asymmetrical –CH_2_ stretching) were observed with increasing microfluidization cycles. These differences may indicate modifications in the arrangement or packing of fatty acid chains within the emulsified oil droplets or changes in the interactions between oil, glycerol, and sodium alginate. Because similar bands appear in pure alginate films (aliphatic CH_2_ group), these peaks likely represent overlapping contributions from both the oil phase and the polysaccharide components.

A modest increase in the intensity of the band at approximately 1737 cm^−1^ (C=O stretching of ester groups in triglycerides) was detected, which became more pronounced with an increasing number of microfluidization cycles. This change may primarily reflect the greater interfacial exposure of ester groups due to reduced droplet size after microfluidization [48].

The band at 1600.10 cm^−1^ (COO^−^ stretching of alginate) showed slight intensity reductions with progressive microfluidization passages. During microfluidic cycles, reductions in particle size increase surface area, which facilitates a higher position to neutralize the COO^−^ stretching of sodium alginate molecules with oil and emulsifiers. This represents a strong interaction between sodium alginate and the oil phase.

The CH_2_ bending band at 1468.66 cm^−1^ displayed small increases in intensity with a higher number of microfluidization cycles. These subtle changes may arise from differences in molecular packing or restricted mobility of lipid chains in the more finely dispersed emulsions, and they may also suggest a slightly higher relative contribution of CH_2_-associated vibrations, which can be consistent with early oxidation-related spectral shifts.

The peak at 1411.52 cm^−1^, assigned to the COO^−^ group of alginate, showed partial splitting into peaks at 1411.52 and 1394.37 cm^−1^. This behavior suggests modifications in the microenvironment of alginate functional groups, likely due to increased interfacial interactions in the nanoemulsion system, rather than formation of new chemical bonds [27,49].

Similarly, the band at 1291.51 cm^−1^ (C–O–C of the alginate pyranose ring) displayed small shifts and variations among treatments, which may indicate altered hydrogen bonding patterns or local rearrangements within the polymer network as the emulsion droplet size decreased [49].

The band at 1171.50 cm^−1^ (C–O stretching and CH_2_ bending in ester groups) also showed slight increases in intensity with microfluidization [50]. These subtle changes may relate to the increased surface area of the dispersed oil phase and improved interaction between emulsifiers and biopolymers.

When comparing the two oils, coconut oil samples showed slightly more pronounced spectral variations than testa oil. This is consistent with the higher content of saturated fatty acids in coconut oil, which may be more sensitive to mechanical shear. In contrast, the natural antioxidants present in testa oil may contribute to its relatively stable spectral profile.

#### 3.7.6. Scanning Electron Microscopic View

**Figure 9:**
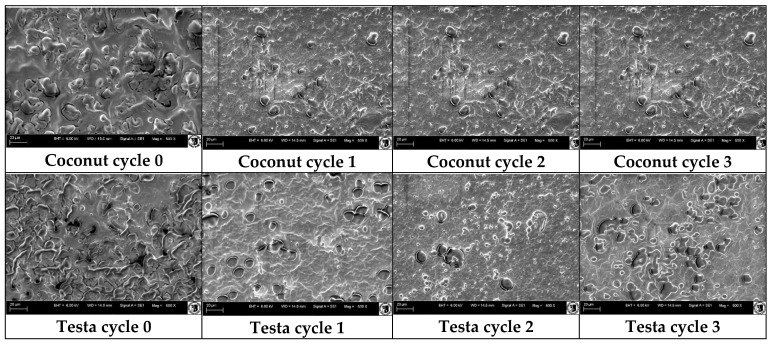
Scanning electronic microscopic view of coconut testa oil added films with microfluidic cycles.

The microstructure of edible films is changed based on the interaction of ingredients that are used for film development. The scanning electron microscopic (SEM) images (×500 magnification) of edible films prepared from emulsions are shown in Figure 9.

SEM images confirmed that the film emulsion exhibited a vesicle-like structure. During the drying process, excess solid material accumulates on the film surface due to solute migration, which may contribute to the formation of a vesicle-like structure. Zhou et al. (2021) identified a vesicle-like structure in scanning electron micrographs of films formulated with cinnamon essential oil, Tween 80, and cassava starch [51].

Wrinkles, pinholes and surface irregularities were observed in the films prepared from the coarse emulsions (without microfluidization), whereas these defects appeared less frequently in films obtained after microfluidization, resulting in visibly more homogeneous structures. The reduction in droplet size with successive microfluidization cycles likely promotes a more compact and uniform arrangement of the sodium alginate network, which in turn may limit the formation of cracks and pinholes during drying. Such a dense polymer–lipid matrix, together with the improved dispersion of oil droplets and emulsifiers, is consistent with the observed decrease in film permeability and the enhancement of mechanical properties reported in this study.

## 4. Conclusions

This study effectively identified the influence of microfluidization and cycle number on the formation and functional properties of nanoemulsions prepared with coconut oil and coconut testa oil. Both microfluidization cycles and oil type significantly affected the characteristics of the nanoemulsion solutions and the resulting edible films. Increasing the number of microfluidization cycles reduced droplet size, which in turn influenced several key attributes of the film-forming solutions, including zeta potential, optical behavior, whiteness index, and creaming stability. These changes further translated into modifications of film properties such as tensile strength, water vapor permeability, and solubility.

Among the two lipid sources, testa oil-based films exhibited higher mechanical strength and lower permeability than coconut oil-based films. These differences may be associated with variations in lipid phase organization and compatibility within the alginate matrix, which can influence the structural integrity and barrier performance of the films. In addition, nanoemulsion-based film systems also provide an efficient platform for incorporating hydrophobic bioactive compounds into edible films, with potential for enhancing the quality and shelf life of selected food products. However, further research is required to examine controlled release behavior, interaction with food surfaces, and performance under practical application conditions.

## Figures and Tables

**Table 1:** Variables and levels in the factorial design required for film-forming nanoemulsion solution.

Treatments	Oil Types	Microfluidic Cycles
C0	Coconut oil	0
C1	Coconut oil	1
C2	Coconut oil	2
C3	Coconut oil	3
T0	Testa oil	0
T1	Testa oil	1
T2	Testa oil	2
T3	Testa oil	3

**Table 2:** Emulsion characteristics: particle size, polydispersity index, zeta potential, pH, EC, whiteness index, thixotropy, loss tangent, creaming index (main and interactional effects).

Emulsion Properties	Main Effects				Interactional Effects	
	Oil Type		Microfluidic Cycles Numer		Oil Type × Microfluidic Cycles	
Particle size [nm]	C	275.20 ± 175.96 ^a^	0	556.18 ± 63.83 ^a^	C0	539.70 ± 92.84 ^a^
					C1	258.90 ± 45.84 ^bc^
			1	267.38 ± 42.54 ^b^	C2	183.80 ± 74.59 ^bcd^
					C3	118.42 ± 31.27 ^d^
	T	288.99 ± 180.16 ^a^	2	177.83 ± 55.71 ^c^	T0	572.65 ± 12.76 ^a^
					T1	275.85 ± 43.94 ^b^
			3	127.01 ± 33.44 ^c^	T2	171.85 ± 39.78 ^bcd^
					T3	135.60 ± 37.87 ^cd^
Polydispersity Index	C	0.59 ± 0.23 ^a^	0	0.79 ± 0.14 ^a^	C0	0.82 ± 0.13 ^a^
					C1	0.68 ± 0.20 ^a^
			1	0.65 ± 0.18 ^a^	C2	0.57 ± 0.04 ^ab^
					C3	0.30 ± 0.07 ^b^
	T	0.55 ± 0.23 ^a^	2	0.55 ± 0.14 ^a^	T0	0.77 ± 0.16 ^a^
					T1	0.62 ± 0.19 ^ab^
			3	0.31 ± 0.07 ^b^	T2	0.52 ± 0.21 ^ab^
					T3	0.31 ± 0.08 ^b^
Zeta potential [mV]	C	−19.36 ± 4.27 ^b^	0	−13.74 ± 2.37 ^a^	C0	−14.39 ± 1.71 ^ab^
					C1	−17.91 ± 1.82 ^abcd^
			1	−17.12 ± 1.59 ^b^	C2	−21.08 ± 1.27 ^cde^
					C3	−24.05 ± 3.79 ^e^
	T	−17.54 ± 3.97 ^a^	2	−19.84 ± 1.72 ^b^	T0	−13.09 ± 3.00 ^a^
					T1	−16.34 ± 0.96 ^abc^
			3	−23.09 ± 3.36 ^c^	T2	−18.60 ± 1.11 ^bcd^
					T3	−22.14 ± 3.09 ^de^
pH	C	5.53 ± 0.18 ^a^	0	5.60 ± 0.20 ^a^	C0	5.58 ± 0.23 ^a^
					C1	5.48 ± 0.25 ^a^
			1	5.53 ± 0.19 ^a^	C2	5.52 ± 0.14 ^a^
					C3	5.54 ± 0.15 ^a^
	T	5.57 ± 0.16 ^a^	2	5.49 ± 0.12 ^a^	T0	5.62 ± 0.19 ^a^
					T1	5.57 ± 0.11 ^a^
			3	5.59 ± 0.18 ^a^	T2	5.46 ± 0.10 ^a^
					T3	5.64 ± 0.22 ^a^
Electrical conductivity [µs/cm]	C	2035.19 ± 102.86 ^a^	0	2039.25 ± 122.41 ^a^	C0	2032.25 ± 119.63 ^a^
					C1	2034.00 ± 110.76 ^a^
			1	2027.25 ± 114.44 ^a^	C2	2037.25 ± 111.09 ^a^
					C3	2037.25 ± 118.12 ^a^
	T	2039.88 ± 109.62 ^a^	2	2042.38 ± 103.72 ^a^	T0	2046.25 ± 143.26 ^a^
					T1	2020.50 ± 134.79 ^a^
			3	2041.25 ± 97.15 ^a^	T2	2047.50 ± 112.66 ^a^
					T3	2045.25 ± 89.60 ^a^
Whiteness Index [WI]	C	73.08 ± 7.82 ^a^	0	66.83 ± 6.33 ^b^	C0	65.86 ± 8.14 ^b^
					C1	73.25 ± 6.48 ^ab^
			1	73.35 ± 5.14 ^ab^	C2	76.08 ± 7.28 ^ab^
					C3	77.12 ± 6.42 ^ab^
	T	74.96 ± 5.93 ^a^	2	77.36 ± 5.14 ^a^	T0	67.80 ± 4.97 ^ab^
					T1	73.46 ± 4.44 ^ab^
			3	78.52 ± 4.69 ^a^	T2	78.64 ± 2.08 ^ab^
					T3	79.92 ± 2.20 ^a^
Thixotropy [Pa.s]	C	1.86 ± 2.94 ^a^	0	7.06 ± 3.50 ^a^	C0	4.031 ± 0.02 ^b^
					C1	3.381 ± 0.79 ^c^
			1	2.45 ± 1.07 ^b^	C2	2.89 ± 0.07 ^d^
					C3	−2.85 ± 0.01 ^f^
	T	1.24 ± 5.88 ^b^	2	0.06 ± 3.26 ^c^	T0	10.09 ± 0.01 ^a^
					T1	1.523 ± 0.03 ^e^
			3	−3.38 ± 0.61 ^d^	T2	−2.76 ± 0.07 ^f^
					T3	−3.91 ± 0.01 ^g^
Loss tangent	C	4.50 ± 4.03 ^a^	0	5.41 ± 5.92 ^a^	C0	10.54 ± 0.07 ^a^
					C1	4.67 ± 0.07 ^b^
			1	2.88 ± 2.07 ^b^	C2	0.82 ± 0.02 ^d^
					C3	1.95 ± 0.80 ^cd^
	T	1.43 ± 1.24 ^b^	2	2.05 ^b^ ± 1.51 ^c^	T0	0.29 ± 0.15 ^d^
					T1	1.09 ± 0.00 ^d^
			3	1.51 ± 0.69 ^c^	T2	3.27 ± 0.90 ^bc^
					T3	1.07 ± 0.05 ^d^
Creaming Index [%]	C	3.83 ± 5.05 ^b^	0	12.19 ± 0.88 ^a^	C0	11.88 ± 1.25 ^a^
					C1	3.44 ± 0.63 ^c^
			1	4.84 ± 1.56 ^b^	C2	0.00 ± 0.00 ^d^
					C3	0.00 ± 0.00 ^d^
	T	4.69 ± 5.35 ^a^	2	0.00 ± 0.00 ^c^	T0	12.50 ± 0.00 ^a^
					T1	6.25 ± 0.00 ^b^
			3	0.00 ± 0.00 ^c^	T2	0.00 ± 0.00 ^d^
					T3	0.00 ± 0.00 ^d^

C: Coconut oil; T: testa oil; 0: coarse emulsion; 1: microfluidic cycle 1; 2: microfluidic cycle 2; 3: microfluidic cycle 3. Values represent means ± standard deviation (*n* = 9). Different superscript letters (a–g) within the same column and within the same emulsion property indicate significant differences (*p* < 0.05) form Tukey posthoc test. Main effects of oil type (C and T) and microfluidic cycles (0, 1, 2, 3), as well as their interaction, are presented separately.

**Table 3:** Edible film characteristics: elongation at break, maximum force for puncture, water vapor permeability and solubility (main and interactional effects).

Film Properties	Main Effects				Interactional Effects	
	Oil Type		Microfluidic Cycles		Oil Type × Microfluidic Cycles	
Elongation at break [%]	C	0.73 ± 0.21 ^b^	0	0.55 ± 0.10 ^c^	C0	0.52 ± 0.10 ^e^
					C1	0.84 ± 0.32 ^bcde^
			1	0.89 ± 0.22 ^b^	C2	0.73 ± 0.04 ^cde^
					C3	0.85 ± 0.08 ^bcd^
	T	0.99 ± 0.30 ^a^	2	0.90 ± 0.19 ^b^	T0	0.59 ± 0.10 ^de^
					T1	0.94 ± 0.07 ^bc^
			3	1.10 ± 0.29 ^a^	T2	1.07 ± 0.06 ^ab^
					T3	1.35 ± 0.17 ^a^
Maximum force for puncture [N]	C	1.90 ± 0.46 ^a^	0	1.34 ± 0.15 ^b^	C0	1.36 ± 0.04 ^b^
					C1	1.78 ± 0.08 ^ab^
			1	1.73 ± 0.19 ^b^	C2	2.31 ± 0.38 ^a^
					C3	2.16 ± 0.44 ^a^
	T	1.92 ± 0.54 ^a^	2	2.30 ± 0.38 ^a^	T0	1.32 ± 0.22 ^b^
					T1	1.68 ± 0.27 ^ab^
			3	2.30 ± 0.38 ^a^	T2	2.28 ± 0.43 ^a^
					T3	2.40 ± 0.32 ^a^
Water vapor permeability [gm^−1^ h^−1^ Pa^−1^]	C	0.61 × 10^−6^ ± 0.92 × 10^−7 a^	0	0.62 × 10^−6^ ± 0.69 × 10^−7 b^	C0	0.66 × 10^−6^ ± 0.53 × 10^−7 ab^
					C1	0.52 × 10−^6^ ± 0.20 × 10^−7 abc^
			1	0.51 × 10^−6^ ± 0461 × 10^−7 a^	C2	0.67 × 10^−6^ ± 0.10 × 10^−6 a^
					C3	0.60 × 10^−6^ ± 0.10 × 10^−6 abc^
	T	0.52 × 10^−6^ ± 0.72 × 10^−7 b^	2	0.61 × 10^−6^ ± 0.11 × 10^−6 b^	T0	0.58 × 10^−6^ ± 0.65 × 10^−7 abc^
					T1	0.50 × 10^−6^ ± 0.66 × 10^−7 bc^
			3	0.53 × 10^−6^ ± 0.11 × 10^−6 ab^	T2	0.54 × 10^−6^ ± 0.66 × 10^−7 abc^
					T3	0.47 × 10^−6^ ± 0.56 × 10^−7 c^
Solubility [%]	C	87.27 ± 5.96 ^b^	0	95.28 ± 4.03 ^a^	C0	92.76 ± 4.18 ^abc^
					C1	89.91 ± 5.26 ^bc^
			1	93.63 ± 5.27 ^a^	C2	86.06 ± 3.74 ^cd^
					C3	80.35 ± 1.75 ^d^
	T	97.11 ± 2.85 ^a^	2	92.36 ± 7.17 ^a^	T0	97.81 ± 1.84 ^a^
					T1	97.34 ± 0.46 ^ab^
			3	87.49 ± 8.37 ^b^	T2	98.65 ± 0.39 ^a^
					T3	94.62 ± 4.96 ^ab^

C: Coconut oil; T: testa oil; 0: coarse emulsion; 1: microfluidic cycle 1; 2: microfluidic cycle 2; 3: microfluidic cycle 3. Values represent means ± standard deviation (*n* = 9). Different superscript letters (a–e) within the same column and within the same film parameter indicate significant differences (*p* < 0.05). Main effects of oil type (C and T) and microfluidic cycles (0, 1, 2, 3), as well as their interaction, are presented separately.

## Data Availability

The original contributions presented in this study are included in the article. Further inquiries can be directed to the corresponding authors.

## Abbreviations

The following abbreviations are used in this manuscript:

CI	Creaming Index
CIE	International Commission on Illumination
EC	Electrical Conductivity
Eq	Equation
FTIR	Fourier Transform Infrared Spectroscopy
G′	Storage Modulus
G″	Loss Modulus
HLB	Hydrophilic–Lipophilic Balance
Mpa	Megapascal
MCFAs	Medium-Chain Fatty Acids
PUFAs	Polyunsaturated Fatty Acids
SEM	Scanning Electron Microscopy
WI	Whiteness Index
WVP	Water Vapor Permeability
WVTR	Water Vapor Transmission Rate
